# Which nurses are victims of bullying: the role of negative affect, core self-evaluations, role conflict and bullying in the nursing staff

**DOI:** 10.1186/s12912-021-00578-3

**Published:** 2021-04-09

**Authors:** Atefeh Homayuni, Zahra Hosseini, Teamur Aghamolaei, Shirin Shahini

**Affiliations:** 1 Health Education and Promotion, Student Research Committee, Faculty of Health, Hormozgan University of Medical Sciences, Bandar Abbas, Iran; 2 Health Education and Promotion, Tobacco and Health Research Center, Hormozgan University of Medical Sciences, Bandar Abbas, Iran; 3 Health Education, Tobacco and Health Research Center, Hormozgan University of Medical Sciences, Bandar Abbas, Iran; 4 Health Education and Promotion, Health School, Hormozgan University of Medical Sciences, Bandar Abbas, Iran

**Keywords:** Bullying, Core self-evaluations, Negative affect, Nurses, Role conflict

## Abstract

**Background:**

Bullying among nurses is a universally recognized problem that has important consequences for nurses, patients and health institutions. This research was conducted with the aim of studying the relationship between role conflict, negative affect and core self-evaluations with bullying in nurses.

**Methods:**

In this cross-sectional study, 329 nurses were selected by census method. Data were collected using PANAS Scale (negative affect), role conflict questionnaire, Core Self-Evaluations Scale (CSES), and the Negative Acts Questionnaire-Revised (NAQ-R). Data were analyzed using t-test, one way analysis of variance, Pearson correlation coefficient and multiple regression analysis with SPSS software (v. 22).

**Results:**

The results showed that there is a significant difference between the mean bullying scores in according to gender and ward of affiliation. The influence of other variables (marital status, education level, years of experience, age group and work position) was not meaningful. Pearson correlation analysis results indicated that there is a significant positive relationship between role conflict (*r* = 0.47) and negative affect (*r* = 0.56) with bullying. Also there is a significant negative relationship between core self-evaluations and bullying (*r* = − 0.39). Moreover, regression analysis results revealed that negative affect, role conflict and gender can predict 44% of bullying variance significantly.

**Conclusions:**

Based on these findings, core self-evaluations, negative affect and role conflict are good factors in predicting bullying among nurses. Consequently, hiring nurses with low negative affect and high core self-evaluations, improving nurses’ self-esteem and self-efficacy and changing workplace conditions in order to reducing role conflict can be useful in reducing workplace bullying.

**Supplementary Information:**

The online version contains supplementary material available at 10.1186/s12912-021-00578-3.

## Background

### Workplace bullying literature

Workplace bullying is considered a serious issue in the nursing profession. The World Health Organization (WHO) has identified the global increase in workplace bullying as a serious threat to nurses’ health and well-being; it has drawn attention to the need for reducing occupational violence as a priority [1]. Workplace bullying occurs when an employee (i.e. the target) is exposed to negative behaviors in the long run against which one feels unable to defend oneself [2]. These negative behaviors may be of a personal (e.g. excessive criticism of personal characteristics, gossip and social deprivation), as well as a work-related nature (e.g. constant belittlement of one’s achievements and unreasonable expectations) or physical intimidation (e.g. aggressive comments and invasion of one’s privacy) [3]**.** Such behaviors are most commonly shown by supervisors or colleagues (i.e. perpetrators) [2]. A study findings suggest that 48% of registered nurses (RNs) admitted to being bullied over the previous 6 months in the workplace, with 35% reported that they had experienced it weekly, and 28% reported that they had experienced it daily [4]. In another study [5], 72% of new nurses reported that they had experienced bullying during previous months. According to the American Nursing Center, workplace bullying is very destructive and may lead to various negative consequences for nurses [6]. Facing bullying is associated with symptoms of depression, anxiety and distress in nurses [7]. Furthermore, workplace bullying can increase stress and decrease job satisfaction in spectator colleagues [8]. These consequences can have important repercussions for health care organizations and the quality of care they provide, as they can reduce the number of human forces at nursing and impose additional and considerable costs in terms of staff replacement and recruitment. Moreover, these consequences reduce RNs’ ability to concentrate, and increase the risks of clinical mistakes and may reduce the safety of patients and the quality of the care they receive [7].

### Antecedents of workplace bullying

Given the worrying consequences of workplace bullying, researchers have identified the antecedents of bullying. So far, these antecedents have been investigated in two separate lines of research, namely work-related factors and those at personal level. Work-related factors refer to aspects of the work environment that require physical and/or psychological skills and effort and are therefore associated with certain physiological and/or psychological costs. Instances of these factors include role stressors, leadership styles and organizational climate. On the other hand, some employees may be more vulnerable to workplace bullying than others due to personality factors. Factors at the personal level relate to aspects of the self and refer to one’s sense of ability to control and influence their environment successfully. Examples of these factors are negative affect, low core self-evaluations and low social skills [9].

#### Role conflict

Role conflict is the existence of conflicting overt and covert demands and requests concerning the roles assigned to individuals which puts people under a lot of pressure and confusion while working [10]. According to workplace hypotheses, poor psychosocial conditions at work such as role ambiguity and conflict might lead to interpersonal conflicts which, if not managed properly, can exacerbate bullying [11].

#### Negative affect

Negative affect refers to the extent in which a person experience negative emotions such as anxiety and depression regardless of the situation [12]. According to Forgas and George [13], individuals’ emotions in the workplace affect the staff’s way of thinking. As described in the affect infusion model, individuals’ affect interact with their cognitive processes exert its effects on different behaviors. This theoretical model attempts to explain how individuals’ affect impacts their ability to encounter with tasks in the workplace, process information and retrieving it, and consequently, affect their judgment and behaviors.

#### Core self-evaluation

Core self-evaluation is another person-related factor that cause bullying. The core self-evaluations construct is viewed as a high-order and stable personality trait and represent individuals’ fundamental assumptions and evaluations about their own worth and competence. It is indicated by four traits: self-esteem, generalized self-efficacy, neuroticism and locus of control. What is common among these traits is known as core self-evaluations [12, 14]. Poor self-view in low core self-evaluations employees make them easy targets for potential bullying perpetrators. Someone who is prone to victimize others may find victimizing these people as easier and more successful, while victimizing those high in core self-evaluations would be more risky and less likely to succeed [12].

### Research framework

According to what was mentioned above, it can be said that personality traits and different workplace conditions may interact in predicting workplace bullying. In Iran, nurse bullying has been studied rarely. In a few studies that have been done, personality traits or aspects of the working environment that cause bullying, have been studied separately. Furthermore, given the importance of bullying issue among nurses and its negative consequences, recognizing and preventing bullying behaviors will play a key role in promoting nurses’ physical and mental health and increasing the productivity, quality and safety of healthcare services. Thus, in the present study we will examine the relationship between role conflict, core self-evaluations and negative affect with bullying. Therefore, the following hypotheses were proposed:

1. Demographic variables can predict bullying among nurses; 2. Role conflict can predict bullying among nurses; 3. Negative affect can predict bullying among nurses; 4. Core self-evaluations can predict bullying among nurses.

## Methods

### Study design and population

A descriptive cross-sectional research design was conducted to examine the relationship between role conflict, negative affect and core self-evaluations with bullying. This study was conducted in both public and private hospitals in Bandar Abbas, south of Iran.

### Sample size and sampling procedure

According to the previous studies, the bullying rate was 31%. Sample size was calculated using the following formula: n = the minimum required sample size, z = level of confidence (1.96), p = parameter for sample calculation (31%), d = margin of error (0.05).

$$ n=\frac{z_{1-a/2}^2\;p\;q}{d^2}=\frac{(1.96)^2\times (0.31)\times (0.69)}{(0.05)^2}=329 $$

Based on this formula, a sample size of 329 anticipated for the study.

Due to the limited statistical population and the need for more detailed information, all nurses were studied through census method. The participants were selected from different wards and different work shifts (morning, evening and night). The inclusion criteria for the sample selection included: (a) at least 1 year of work experience as a nurse in hospital and (b) willingness to participate in research. The exclusion criteria were: (a) less than 1 year’s nursing experience and (b) having time pressure for participation.

Prior to data collection, the researcher gained the required permission from the university and obtained the institutional consent from hospitals’ managements. Then, the researcher explained in detail about the purpose of the study, advantages and how to fill out the questionnaires. They were assured that they could withdraw from the study at any time. The questionnaires were given to those who volunteered to participate in the study. To ensure voluntary participation in the study, participants were asked to give their consent. All participants were given consent forms to sign.

### Study instrument

We used self-administered questionnaires to collect the data. The questionnaires were demographic characteristics questionnaire, positive and negative affect scale, role conflict questionnaire, core self-evaluations scale and negative act questionnaire.

Demographic characteristics assessed were gender, marital status, educational level, years of experience, age group, ward of affiliation and work position.

### Positive and negative affect scale

To measure the negative affect, the negative affectivity (NA) scale from the well-known PANAS instrument was used. It is a 20-item self-rating instrument developed by Watson et al. [15], which measured negative and positive affect. Each subscale consisted of 10 items. All items were scored on a 5-point Likert scale ranging from very low (score 1) to very high (score 5). Overall scores for each subscale was 10 to 50 points. Internal compatibility coefficient (alpha) is 0.87 for the negative affect subscale [15]. In the present study, Cronbach’s alpha and Split-half coefficient for the negative affect subscale were 0.91 and 0.92, respectively.

### Role conflict questionnaire

Role conflict was measured by 8 questions through Rizzo’s role conflict and ambiguity questionnaire [16]. It was a 4 point Likert scale ranging from “1 = completely true” to “4= not at all”. Overall scores ranged from 8 to 32 points whereby the higher score indicated the lower role conflict. In the present study, Cronbach’s alpha and Split-half coefficient of this questionnaire were 0.87 and 0.81, respectively.

### Core self-evaluations scale

The Core Self-Evaluations Scale (CSES) was used to measure core self-evaluations. This scale was developed by Judge et al. [17]. It is a 12- item instrument scored on a 5-point Likert scale from “strongly disagree” (1) to “strongly agree” (5). A higher score would indicate a higher core self-evaluations. Items 2, 4, 6, 8, 10 and 12 were reversely scored. Judge et al. [17] estimated the reliability of this questionnaire via Cronbach’s alpha and split-half method and reported it respectively 0.78 and 0.86. In the present study, Cronbach’s alpha and Split-half coefficient of this questionnaire were 0.72 and 0.74, respectively.

### Bullying questionnaire

Bullying behaviors were rated on the revised version of negative act questionnaire (NAQ-R) [3]. This measurement instrument aimed to measure the degree of bullying and harassment at work. The participants were supposed to recall their last 6 working months and express their agreement or disagreement on a 5-point Likert scale: never (score 1), sometimes (score 2), once a month (score 3), once a week (score 4) and every day (score 5). Overall scores ranged from 22 to 120. A score of 22 means that the participant has not been exposed to any form of bullying during the last 6 months and a higher score would indicate a longer experience of bullying and harassment. In an exploratory factor analysis of the questionnaire, Einarsen et al. [3] found three constituent factors: work-related bullying (7 items), person-related bullying (12 items) and physical intimidation (3 items). These three factors were confirmed by a confirmatory factor analysis. To validate the questionnaire, Cronbach’s alpha was used, which was estimated at 0.90 for the whole scale. In the present study, Cronbach’s alpha and Split-half coefficient of this questionnaire were 0.92 and 0.75, respectively.

### Data analysis

Data were analyzed using the Statistical Package for the Social Sciences (SPSS) version 22. The demographic characteristics of the respondents were described using descriptive statistics including frequency and percentage. Means and standard deviations were calculated for continuous variables. We used the participants’ responses to items of the same domain in the scale to replace missing data. Differences between the groups were tested by t-test and one way analysis of variance (ANOVA). Pearson correlation analysis was conducted to explore the relationship between variables. Hierarchical multiple regression was used to assess the ability of predictor variables to predict bullying. The level of significance was considered to be 95% (*p* < 0/05).

## Results

### Socio-demographic characteristics

Among 329 distributed questionnaires, a total number of 321 were completed and returned. The participants’ demographic information is summarized here: 12.5% of the participants were male and 87.5% were female. In relation to their marital status, 73.8% were married, while 24.6% were single. In connection with respondents’ level of education, the majority of nurses had Bachelor’s degree as their highest qualifications (84.5%). About half of them (43.9%) were between the ages of 30 and 39 years. The results further indicate that 82.2% of the participants participated as a nurse. The majority of participants (38.6%) had more than 10 years’ experience of nursing service (Table 1).

**Table 1:** Participants’ characteristics (*N* = 321)

Characteristics	Categories	N (%)
Gender	Male	40 (12.5)
	Female	281 (87.5)
Marital status	Single	79 (24.6)
	Married	237 (73.8)
	Divorced	5 (1.6)
Education level	Diploma	19 (5.9)
	Associate degree	3 (0.9)
	Bachelor’s degree	271 (84.5)
	Master’s degree and higher	28 (8.7)
Years of experience	< 3 years	51 (15.9)
	3–5 years	48 (15)
	5–10 years	98 (30.5)
	> 10 years	124 (38.6)
Age group	20–29 years	115 (35.9)
	30–39 years	141 (43.9)
	≥40 years	65 (20.2)
Ward of affiliation	Children	13 (4.04)
	Adults	29 (9.03)
	ICU	57 (17.75)
	Internal ward	47 (14.64)
	Thalassemia and dialysis	23 (7.2)
	CCU	35 (10.9)
	NICU	20 (6.23)
	Neurology	45 (14.01)
	Operating room and maternity ward	52 (16.2)
Work position	Matron	16 (5)
	Nurse	264 (82.2)
	Others	41 (12.8)

The results of t-test showed that there is a significant difference between the mean bullying scores in male and female nurses (df = 317, t = 4.118, *p* < 0.000). Mean score on bullying was significantly higher for male than female nurses (Table 2).

**Table 2:** T-test results to compare the mean bullying scores in terms of gender

variable		Mean ± Standard Deviation	T	df	Sig
Gender	Male	46.65 ± 13.19	4.118	317	0.000
	Female	37.79 ± 12.66			

One-Way Anova results showed that there aren’t significant differences in bullying mean scores according to marital status, education level, years of experience, age group and work position, but nurses in different wards experienced varying amounts of bullying. Nurses who worked at Operating room and maternity ward experienced more bullying than nurses who worked at other wards. The score for bullying was significantly lower for nurses who worked at CCU (Table 3).

**Table 3:** One-Way Anova results to compare the mean bullying scores in terms of demographic variables

variable		Mean ± Standard Deviation	F	Sig
Marital status	Single	39.07 ± 12.54	0.912	0.403
	Married	38.67 ± 12.94		
	Divorced	46.6 ± 24.20		
Education level	Diploma	39.58 ± 12.32	0.701	0.552
	Associate degree	43 ± 15.39		
	Bachelor’s degree	39.14 ± 13.07		
	Master’s degree and higher	35.71 ± 13.21		
Years of experience	< 3 years	40.37 ± 12.64	0.423	0.737
	3–5 years	38.27 ± 11.12		
	5–10 years	38.03 ± 12.52		
	> 10 years	39.23 ± 14.34		
Age group	20–29 years	39.84 ± 11.59	0.576	0.563
	30–39 years	38.08 ± 13.77		
	≥40 years	39 ± 13.94		
Ward of affiliation	Children	38.23 ± 9.98	2.263	0.023
	Adults	40.03 ± 14.81		
	ICU	39.59 ± 13.33		
	Internal ward	35.65 ± 9.63		
	Thalassemia and dialysis	37.74 ± 13.56		
	CCU	34.86 ± 7.63		
	NICU	40.7 ± 10.42		
	Neurology	37.27 ± 14.51		
	Operating room and maternity ward	44.52 ± 15.64		
Work position	Matron	31.73 ± 9.97	2.397	0.093
	Nurse	39.25 ± 12.97		
	Others	39.24 ± 14		

The mean and standard deviation of the variables, as well as their correlation with each other are presented in Table 4.

**Table 4:** Mean, standard deviation & correlation coefficients of study variables

Variable	Mean	Standard deviation	1	2	3	4	5	6	7
**Bullying**	38.9	13.04	1	0.94^**^	0.86^**^	0.64^**^	0.56^**^	−0.39^**^	0.47^**^
**Person-related bullying**	18.9	7.39	0.94^**^	1	0.66^**^	0.61^**^	0.52^**^	−0.38^**^	0.46^**^
**Work-related bullying**	15.9	5.64	0.86^**^	0.66^**^	1	0.36^**^	0.53^**^	−0.33^**^	0.44^**^
**Physical intimidation**	4.24	1.62	0.64^**^	0.61^**^	0.36^**^	1	0.33^**^	−0.22^**^	0.32^**^
**Negative affect**	18.58	7.62	0.56^**^	0.52^**^	0.53^**^	0.33^**^	1	−0.51^**^	0.42^**^
**Core self-evaluations**	42.12	5.42	−0.39^**^	−0.38^**^	− 0.33^**^	−0.22^**^	− 0.51^**^	1	− 0.32^**^
**Role conflict**	18.55	5.89	0.47^**^	0.46^**^	0.44^**^	0.32^**^	0.42^**^	−0.32^**^	1

^**^P < 0.01

As the results showed, the mean and standard deviation scores of core self-evaluations were (42.12 ± 5.42), role conflict (18.55 ± 5.89), negative affect (18.58 ± 7.62), bullying (38.9 ± 13.04), person-related bullying (18.9 ± 7.39), work-related bullying (15.9 ± 5.64) and physical intimidation (4.24 ± 1.62).

Results of Pearson correlation indicated there were significant relationships between the independent variables (negative affect, core self-evaluations and role conflict) with bullying (*p* < 0.01). While core self-evaluations was negatively correlated with dependent variable (bullying and its dimensions), other variables (role conflict and negative affect) were found to be positively associated with bullying and its dimensions (p < 0.01).

A hierarchical multiple regression ascertained how much variance in bullying could be accounted for by independent variables (Table 5).

**Table 5:** Results of multiple hierarchical regression analysis in predicting bullying through predictor variables

	Variables	B	S.E	β	T	***P*** Value	R	R^**2**^	∆R^**2**^
Model 1	Negative affect	.95	.08	.56	11.92	.000	.56	.31	–
Model 2	Core self-evaluations	.83	.09	.49	9.02	000	.58	.33	.02
		−.34	.13	−.14	−2.62	.009			
Model 3	Role conflict	.65	.09	.38	7.12	.000	.64	.41	.08
		−.23	.12	−.09	−1.91	.057			
		.69	.11	.31	6.41	.000			
Model 4	Gender	65.	0.09	.38	7.27	000.	.65	.43	.02
		−.22	.12	−.09	−1.81	.072			
		.64	.11	.29	5.9	.000			
		−5.57	1.74	−.14	−3.12	.002			
Model 5	Age groups	.65	.09	.38	7.22	.000	.66	.44	.01
		−.22	.12	−.09	−1.85	.065			
		.65	.11	.29	6.07	.000			
		−5.05	1.79	−.13	−2.83	.005			
		−1.02	.79	−.06	−1.29	.199			

Negative affect, core self-evaluations, role conflict, gender and age group were entered the regression equation in the first, second, third, fourth and last step, respectively. 31% of variances of bullying was accounted for by negative affect in the first step and by core self-evaluations in step 2 as 2%. An additional 8% of the variance in bullying was explained by the addition of role conflict in step 3. Finally, gender was accounted for an additional 2% variance in bullying, and age group was accounted for an additional 1% variance in bullying. The results showed that the multiple correlation of the variables involved in bullying prediction is *R* = 0.66 and its square is *R*^2^ = 0.44. On the other hand, negative affect, role conflict and gender can predict 44% of the total variance in bullying.

## Discussion

This study examined the relationship between demographic variables, role conflict, negative affect and core self-evaluations with bullying. The results showed that there is a significant difference between the mean bullying scores in according to gender and ward of affiliation. Results indicated that there are significant positive relationship between role conflict and negative affect with bullying and there is a significant negative relationship between core self-evaluations with bullying.

### The relationship between demographic characteristics with bullying

The results showed that men are more exposed to bullying behaviors. In other words, men are the most bullied in our study. Also, the study reported that nurses in different wards experience different levels of bullying behaviors. Several researchers [18–20] reported that women become more a target of bullying than men at work. However, others [21, 22] found and reported minor or no difference across gender. The findings described above indicated that there aren’t significant differences in bullying mean scores according to marital status, education level, years of experience, age group and work position. This finding is supported by the findings from different studies conducted by Yildirim [23], Chen et al. [24], Yildirim and Yildirim [25], and Yavuzer and Civilidg [26]. Findings from a cross-sectional study conducted in Turkey by Yildirim [23] showed that there were no significant differences between position and educational level in regard to workplace bullying. This research also revealed that bullying was positively correlated with work overload and total years of work in nursing. Bullying and nurses’ age showed to be negatively correlated. Contrary to this finding, Duru et al. [27] showed that the workplace bullying perception scores decreased with increases in the employees’ age and increased with being divorced. This score was found to be higher in the 20–29-years-old age group.

Moreover, Einarsen and Skogstad [22] found that older workers are at a higher risk of victimization than younger ones. Finally, unlike our study, Sahin et al. [28] reported that the single staff were at a higher risk of workplace bullying.

### The relationship between role conflict with bullying

This study found that role conflict can predict bullying. The current finding is similar with findings from other studies conducted by Van den Brande et al. [9], Trepanier et al. [7], Bowling and Beehr [29], Balducci et al. [30] and Mathisen et al. [31]. In a longitudinal research among 234 employees of a health service agency including nurses, physicians and administration employees, Balducci et al. [30] found that role conflict predicted both being bullied and bullying enactment over time [7]. Studies shows that bullying occurs in the presence of negative job features and the absence of positive ones. Job features are the main aspects of employees’ daily duties. Negative job features (e.g. high work overload, role conflict and emotional demands) require constant effort and are associated with certain physical and psychological costs. In the nursing profession, the association between job features (i.e. negative features and the absence of positive features) to some extent explain why nurses are more vulnerable to bullying. Operational constraints are increasing in healthcare institutions and they are under significant pressure to enhance the efficiency and quality of their services. This often lead to the constant monitoring of nursing activities (i.e. the lack of occupational control) as well as increasing workloads, longer work hours and pressure for the nursing teams with fewer human forces. These conditions all would pave the way for bullying in the workplace [7]. Moreover, nurses are in the closest contact with patients and are exposed much more often to patients’ suffering, disease and death. They almost always have to work in a stressful condition with a high workload. In addition, a nurse works in a three-shift system. Such a work system may cause uncertainties in tasks or responsibilities among nurses. In such conditions, role conflict between nurses can naturally grow and lead to workplace bullying [6]. Thus, it can be said that role conflict, as a negative job feature, can lead to bullying in the workplace.

### Relationships between negative affect and core self-evaluations with bullying

In this study it was also demonstrated that negative affect and core self-evaluations predicted bullying. These findings are in consistent with previous studies conducted by Bowling and Beehr [29], Rodwell and Demir [32], Bowling et al. [12], Matthiesen and Einarsen [33] and Podsiadly and Gamian-Wilk [34]. In the majority of theoretical models, personality of the bullying victim plays a major and key role in explaining victimization from bullying. It has been argued that people’s tendencies may trigger negative behaviors on the part of colleagues and employers. From this perspective, employees who have certain traits or those highly vulnerable may violate the expectations and norms at work and, thus, aggravating others. Different studies show that, compared to the oppressors and non-victims, the target group obtains higher scores on neuroticism, depression and negative affect and lower scores on emotional stability and self-esteem and a higher score on temperamental emotional reactivity [34]. In a meta-analysis, Bowling and Beehr [29] examined the victim’s personality as a predictor of victimization. They found that victim negative affect (*p* = 0.25) and victim self-esteem (*p* = − 0.21) were associated to victimization; the victim positive affect (*p* = − 0.09) showed a relatively weak correlation with victimization. There are two mechanisms through which a potential victim’s personality can elicit bullying. First, this person might act offensively and tempt anyone with a potential for victimizing others. Negative affect could be associated with such offensive behaviors. The negative perspectives of those with a high negative affect might lead them to talk negatively about the workplace and colleagues, which can be annoying to others [12]. The second mechanism through which this victim’s personality can lead to victimization is that the target appear vulnerable to victimization. Vulnerability is the more likely mechanism for employees with low core self-evaluations. Core self-evaluations encompasses an individual’s beliefs about one’s competencies (life control) and qualifications including performance, coping strategies and achievement [35]. Core self-evaluations is defined as one’s beliefs about their ability to interact successfully with their surrounding environment through changing and correcting their behaviors and external events. People with negative core self-evaluations believe that their actions are useless. They might feel themselves scarcely capable of improving conditions. Thus, they are more prone to experience negative affect and emotions [36]. Individuals with negative core self-evaluations underestimate their capabilities in comparison to others. They focus on their failures and defects and perceive themselves as a victim to environment [37]. Low self-view in these employees makes potential perpetrators see them as easy targets. Someone who is prone to victimize others might find such people an easy target and stands higher chances of success. However, victimizing others with a higher core self-evaluations might be more risky and stand lower chances of success [12].

### Multiple relationships between independent variables with bullying

Finally, the hierarchical regression analysis showed negative affect, role conflict and gender can significantly predict bullying. The higher prevalence of bullying among nurses is probably due to the characteristics of nursing profession as a stressful job. Researchers have showed that high job stress or conflict, high work load and low autonomy are significantly correlated with a higher level of bullying at workplace [6]. The possible explanation for the prediction power of negative affect in workplace bullying is that the negative affect might act as a potential perceptual bias, whereby employees with higher level of negative affect perceive behaviors as more personal than they actually are and, thus, report more cases of bullying at workplace [32].

#### Study limitations

This study has some limitations that should be noted. First, as this study has a correlational design and it did not seek to establish cause and effect, drawing any causal conclusions from the results is not possible. In order to show the causality, it is recommended that further studies adopt longitudinal or experimental designs. In addition, this study relied on self-report measures. Incorporating multimethod approaches may increase the validity of the findings. Second, the respondents were asked only if they felt subjected to bullying behaviors at the workplace. In future studies we can examine if participants had acted as perpetrators. Third, the respondents were asked about a sensitive topic (bullying), an issue that may have led them to respond with denial or social desirability. The result may be valid to the extent that respondents answered the questions in a honest way. Finally, we know that there are other factors related to workplace bullying that have not been taken into account here, and should be considered by researchers in future studies.

The present study also has several strengths. All instruments used in the present study are well validated and have acceptable psychometric properties. Participants were included in the study from different hospitals including public and private hospitals.

## Conclusions

Our findings showed that particular personality variables (i.e., negative affect and core self-evaluations) and work-related factors (i.e., role conflict) can predict the exposure to bullying behaviors. It could therefore be beneficial to assess negative affect and core self -evaluations in personnel selection especially for highly stressful and demanding jobs such as nursing. These personality factors are relatively stable constructs and are malleable due to trainings. Additionally, hospitals can provide clear and specific job descriptions for nurses. This action will largely prevent role conflict. Finally, it is noteworthy that though the present findings showed that the victim’s personality affects the perpetration of bullying, this finding should not be used to blame victims. Hospitals should create a highly competent work environment and implement anti-bullying policies to support their staff at workplace. They can also develop a system for reporting all events related to bullying at hospital.

## Supplementary Information

**Additional file 1.**

**Additional file 2.**

## Data Availability

The datasets used and/or analyzed during the current study are available from the corresponding author on reasonable request.

## Abbreviations

CSES: Core Self-Evaluations Scale
NAQ-R: Negative Acts Questionnaire-Revised
PANAS: Positive and Negative Affect Scale
RNs: Registered nurses
WHO: World Health Organization
