# Supplementary material for: Which nurses are victims of bullying: the role of negative affect, core self-evaluations, role conflict and bullying in the nursing staff
Source: BMC Nurs. 2021 Apr 9;20:57. doi: 10.1186/s12912-021-00578-3 (PMC8034186; doi:10.1186/s12912-021-00578-3)
Supplement: Supplementary file 1 — Additional file 1. [file 12912_2021_578_MOESM1_ESM.docx]

**Additional Files (questionnaires)**

***1.Role Conflict Questionnaire***

1. I have to do things that should be done differently under different conditions.
2. I receive an assignment without the manpower to complete it.
3. I have to buck a rule or policy in order to carry out an assignment.
4. I work with two or more groups who operate quite differently.
5. I receive incompatible requests from two or more people.
6. I do things that are apt to be accepted by one person and not accepted by others.
7. I receive an assignment without adequate resources and materials to execute it.
8. I work on unnecessary things.

***2. The Core Self -Evaluations Scale (CSES)***

Instructions: Below are several statements about you with which you may agree or disagree. Using the response scale below, indicate your agreement or disagreement with each item by placing the appropriate number on the line preceding that item.

1: strongly disagree

2: disagree

3: neutral

4: agree

5: strongly agree

1……I am confident I get the success I deserve in life.

2……Sometimes I feel depressed. *(r)*

3……When I try, I generally succeed.

4…….Sometimes when I fail I feel worthless. *(r)*

*5……...*I complete tasks successfully.

6……..Sometimes, I do not feel in control of my work. *(r)*

7……. Overall, I am satisfied with myself.

8……...I am filled with doubts about my competence. (*r*)

9……...I determine what will happen in my life.

10…….I do not feel in control of my success in my career. (*r*)

11…….I am capable of coping with most of my problems.

12…… There are times when things look pretty bleak and hopeless to me. (*r*)

(R=REVERSE-SCORED.)
