# Supplementary material for: Which nurses are victims of bullying: the role of negative affect, core self-evaluations, role conflict and bullying in the nursing staff
Source: BMC Nurs. 2021 Apr 9;20:57. doi: 10.1186/s12912-021-00578-3 (PMC8034186; doi:10.1186/s12912-021-00578-3)
Supplement: Supplementary file 2 — Additional file 2. [file 12912_2021_578_MOESM2_ESM.docx]

**1.The PANAS**

To measure the negative affect, the well-known PANAS instrument was used. This scale consists of a number of words that describe different feelings and emotions. Read each item and then mark the appropriate answer in the space next to that word. Indicate to what extent [INSERT APPROPRIATE TIME INSTRUCTIONS HERE]. Use the following scale to record your answers

1: very slightly or not at all

2: a little

3: moderately

4: quite a bit

5: extremely

| **Positive affect** | **Negative affect** |
| --- | --- |
| alert | irritable |
| interested | ashamed |
| inspired | distressed |
| excited | upset |
| determined | guilty |
| strong | scared |
| attentive | hostile |
| active | jittery |
| enthusiastic | afraid |
| proud | nervous |

**2.Role Conflict Questionnaire**

1. I have to do things that should be done differently under different conditions.
2. I receive an assignment without the manpower to complete it.
3. I have to buck a rule or policy in order to carry out an assignment.
4. I work with two or more groups who operate quite differently.
5. I receive incompatible requests from two or more people.
6. I do things that are apt to be accepted by one person and not accepted by others.
7. I receive an assignment without adequate resources and materials to execute it.
8. I work on unnecessary things.

**3. The Core Self -Evaluations Scale (CSES)**

**Instructions:** Below are several statements about you with which you may agree or disagree. Using the response scale below, indicate your agreement or disagreement with each item by placing the appropriate number on the line preceding that item.

1: strongly disagree

2: disagree

3: neutral

4: agree

5: strongly agree

1……I am confident I get the success I deserve in life.

2……Sometimes I feel depressed. ***(r)***

3……When I try, I generally succeed.

4…….Sometimes when I fail I feel worthless. ***(r)***

*5……...*I complete tasks successfully.

6……..Sometimes, I do not feel in control of my work. ***(r)***

7……. Overall, I am satisfied with myself.

8……...I am filled with doubts about my competence. **(r)**

9……...I determine what will happen in my life.

10…….I do not feel in control of my success in my career. **(r)**

11…….I am capable of coping with most of my problems.

12…… There are times when things look pretty bleak and hopeless to me. **(r)**

(R=REVERSE-SCORED.)

**4. The Revised version of Negative Act Questionnaire (NAQ-R)**

(The version of the NAQ-R tested in this study has 22 items, measuring exposure to bullying within the last 6 months, with the response alternatives: ‘‘Never,’’ ‘‘Now and then,’’ ‘‘Monthly,’’ ‘‘Weekly’’ and ‘‘Daily’).

1.Someone withholding information which affects your performance.

2. Being ordered to do work below your level of competence.

3. Having your opinions ignored.

4. Being given tasks with unreasonable deadlines.

5. Excessive monitoring of your work.

6. Pressure not to claim something to which by right you are entitled (e.g. sick leave, holiday entitlement, travel expenses)

7. Being exposed to an unmanageable workload.

8. Being humiliated or ridiculed in connection with your work.

9. Having key areas of responsibility removed or replaced with more trivial or unpleasant tasks 10. Spreading of gossip and rumours about you.

11. Being ignored or excluded.

12. Having insulting or offensive remarks made about your person, attitudes or your private life

13. Hints or signals from others that you should quit your job.

14. Repeated reminders of your errors or mistakes.

15. Being ignored or facing a hostile reaction when you approach.

16. Persistent criticism of your errors or mistakes.

17. Practical jokes carried out by people you don’t get along with.

18. Having allegations made against you.

19. Being the subject of excessive teasing and sarcasm.

20. Being shouted at or being the target of spontaneous anger.

21. Intimidating behaviors such as finger-pointing, invasion of personal space, shoving, blocking your way.

22. Threats of violence or physical abuse or actual abuse
